# Continuous Live-Cell Culture Imaging and Single-Cell Tracking by Computational Lensfree LED Microscopy

**DOI:** 10.3390/s19051234

**Published:** 2019-03-11

**Authors:** Gregor Scholz, Shinta Mariana, Agus Budi Dharmawan, Iqbal Syamsu, Philipp Hörmann, Carsten Reuse, Jana Hartmann, Karsten Hiller, Joan Daniel Prades, Hutomo Suryo Wasisto, Andreas Waag

**Affiliations:** 1 Institute of Semiconductor Technology (IHT), Technische Universität Braunschweig, 38106 Braunschweig, Germany; s.mariana@tu-braunschweig.de (S.M.); a.dharmawan@tu-braunschweig.de (A.B.D.); i.syamsu@tu-braunschweig.de (I.S.); jana.hartmann@tu-braunschweig.de (J.H.); a.waag@tu-braunschweig.de (A.W.); 2 Laboratory for Emerging Nanometrology (LENA), Technische Universität Braunschweig, 38106 Braunschweig, Germany; 3 Faculty of Information Technology, Universitas Tarumanagara, Jakarta 11440, Indonesia; 4 Research Center for Electronics and Telecommunication, Indonesian Institute of Sciences (LIPI), Bandung 40135, Indonesia; 5 Institute for Biochemistry, Biotechnology and Bioinformatics, Technische Universität Braunschweig, 38106 Braunschweig, Germany; p.hoermann@tu-braunschweig.de (P.H.); c.reuse@tu-braunschweig.de (C.R.); karsten.hiller@tu-braunschweig.de (K.H.); 6 MIND-IN^2^UB, Department of Electronic and Biomedical Engineering, University of Barcelona, 08028 Barcelona, Spain; dprades@el.ub.edu

**Keywords:** lensless holographic microscopy, LED, Complementary Metal-Oxide Semiconductor (CMOS) image sensor, cell culture, cell imaging, cell counting

## Abstract

Continuous cell culture monitoring as a way of investigating growth, proliferation, and kinetics of biological experiments is in high demand. However, commercially available solutions are typically expensive and large in size. Digital inline-holographic microscopes (DIHM) can provide a cost-effective alternative to conventional microscopes, bridging the gap towards live-cell culture imaging. In this work, a DIHM is built from inexpensive components and applied to different cell cultures. The images are reconstructed by computational methods and the data are analyzed with particle detection and tracking methods. Counting of cells as well as movement tracking of living cells is demonstrated, showing the feasibility of using a field-portable DIHM for basic cell culture investigation and bringing about the potential to deeply understand cell motility.

## 1. Introduction

Continuous cell culture monitoring is of high interest for the investigation of growth, proliferation, and kinetics of biological samples. In addition to biochemical sensors that provide information about the presence and quantity of metabolism products [1], cell culture imaging provides information on cell count, cell shape, and cell movement, and allows the tracking of individual cells within a culture. However, continuous imaging of cell cultures is challenged both by the need for high image quality and compatibility with the cultivation environment. Therefore, culture observation is predominantly done by end-point investigation. The continuous availability of experiments is traded in for stable cultivation conditions and highly sophisticated assessment at the end of cell cultivation. However, observations of proliferation and differentiation kinetics could lead to a more thorough understanding of biological processes in cell cultures, and they are therefore highly demanded [2]. Even though some high-end microscopes, which could be integrated into a cell incubation environment or have an integrated incubator unit, exist, those devices are highly priced and therefore not suitable for large scale or long-term investigations. Furthermore, small and inexpensive microscopes that could be hooked up to all individual samples for an extensive period are more desirable, especially in fields where high-end laboratories are not available.

Recently, digital inline-holographic microscopes (DIHM) based on light-emitting diode (LED) illumination have gained attention for providing a small and inexpensive but capable microscopy solution [3,4,5,6]. Due to their not relying on lenses and essentially consisting only of a light source and an image sensor, they can be built in a very cost effective manner [7]. Illumination by LED can provide a sufficiently high level of spatial and spectral coherency [8] that leads to the formation of diffraction images of the sample on the sensor. The computational reconstruction of the object wave front reveals both amplitude and phase images, which lead to additional insights into the sample. For organic samples especially, this method can make label-free investigation of cellular compartments feasible that otherwise would require staining [9]. Furthermore, computational image enhancements can be performed to overcome the limitations of the light source and the image sensor [10].

In this work, a compact and inexpensive DIHM based on a 5 megapixel complementary metal-oxide semiconductor (CMOS) image sensor and a multicolor LED was built for investigation of biological samples inside petri dishes and well plates. The multicolor LED and the image sensor being capable of video frame rate imaging allows for high flexibility in application. The choice of an LED light source avoids the speckle noise produced by laser light sources and allows for adaptation to cell cultures and media of different spectral absorbances. The image sensor provides a large maximum field-of-view of 5.7 × 4.3 mm^2^ and allows for both time-lapsed investigations and real-time imaging. Furthermore, the housing of the microscope was built by 3D printing, allowing for it being adapted to a variety of sample form factors. Image reconstruction was carried out via an angular spectrum approach on a regular PC. In order to show its capability regarding imaging and monitoring of live-cell cultures, the setup was applied to both cultures of neuroblastoma and *Prorocentrum minimum (P. minimum)*. Cell registration and counting as well as the tracking of moving cells was carried out. Neuroblastoma cultures acted both as a model culture for cell counting and as a way to distinguish between different cells via simple image filtering criteria. *P. minimum*, being a harmful alga that occurs in coastal water worldwide, served as a sample for proof-of-concept concentration measurement and single-cell tracking.

## 2. Materials and Methods

### 2.1. Cell Cultures

#### 2.1.1. Neuroblastoma Culture

**Figure 1:**
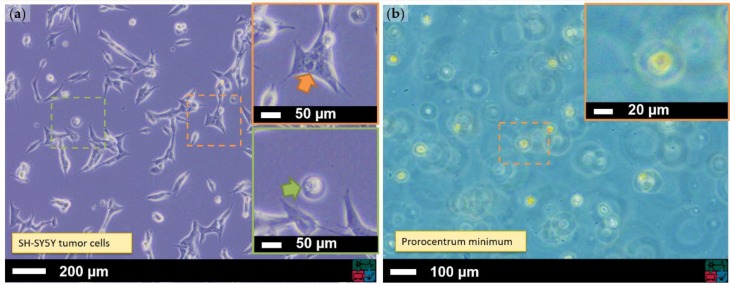
Micrographs of the cell culture samples that have been investigated: (**a**) SH-SY5Y neuroblastoma cell culture with N-type cell (upper inset) and S-type cell (lower inset); and (**b**) *Prorocentrum minimum* cell culture with singe cell (inset). The images where taken by an inverted optical microscope.

In this work, the neuroblastoma cell line SH-SY5Y (ATCC^®^ CRL-2266, Figure 1a) was investigated. This cell line is a sub clone of the original SK-N-SH cell line that originates from human bone marrow [11]. Due to its ability to be differentiated into neuronal cells [12], it is an easier and less expensive alternative to primary neuronal cultures. Because of their cancerous origin, these immortalized cells can be easily expanded and ethical issues can be circumvented.

The undifferentiated cells were seeded in 12-well cell culture plates and incubated at 37 °C and 5% CO_2_. The viable cells are adherent and show an irregular shape (Figure 1a, Inset 1), while non-viable cells are not able to adhere to the surface (Figure 1a, Inset 2). During experiments, two different cell seeding densities were investigated.

#### 2.1.2. Prorocentrum Minimum Culture

*Prorocentrum minimum* (Figure 1b) is a eukaryotic single-cell alga which belongs systematically to the superclass of Dinoflagellate. These complex organisms exhibit a genome which is up to two times larger than the human genome [13]. They are able to grow phototrophically by using light, H_2_O, and CO_2_ to generate carbohydrates or heterotrophically by digesting other small organisms [14]. *P. minimum* has a triangular oval-round shape with a typical length of 17–25 µm, a width of 14–23 µm, and two flagellar coming out of its top point [15]. Therefore, *P. minimum* is capable of actively moving through a medium. Interest in this organism increased when events with a rapid biomass increase, also known as bloom forming, became evident. Associated toxin accumulation can lead to shellfish poisoning [16].

*P. minimum* cultures were grown at 20 °C under a day/night cycle of 12/12 h. The cultures revealed reproducible exponential growth in an artificial seawater medium and samples were taken during the exponential growth phase.

In this investigation, three cultures with different cell densities were prepared and placed in petri dishes. In order to prevent condensed water droplets from obstructing the holographic image, the lid was removed for the measurement. In addition, drops of the culture were taken and placed on a microscope slide with a cover slip on top.

### 2.2. Microscopy Setup

**Figure 2:**
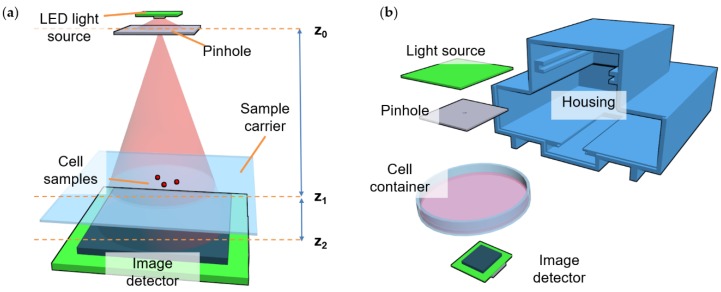
Schemes of the digital inline-holographic microscope (DIHM): (**a**) Principle sketch illustrating the position of the light-emitting diode (LED) light source, cell sample, and image sensor relative to each other; and (**b**) scheme of the full system, including a 3D-printed housing and a petri dish.

A DIHM based on LED illumination and a CMOS image sensor was built. A schematic drawing of the setup is shown in Figure 2a. The used LED light source was a multi-color SMD LED (KAA-3528RGBS-11, Kingbright, Taipei, Taiwan) showing illumination peaks at 466 nm, 517 nm, and 629 nm. The light source was spatially filtered by a laser-machined 90 µm pinhole to increase the spatial coherence of the sample illumination. The biological sample was placed on top of an image sensor (MT9P031, ON Semiconductor, Phoenix, AZ, USA) with a maximum resolution of 2592 × 1944 pixels and a pixel pitch of 2.2 µm. The sensor was interfaced by a Basler Dart controller board and connected to a PC by a USB 3.0, allowing for a 7 frames per second (fps) image acquisition of the full frame. The controlling of the light source was performed by a microcontroller (ATmega328P, Microchip Technology, Chandler, AZ, USA) that was linked to the measurement PC via a serial interface.

Control of the image acquisition was performed by in-house written software, which enabled both video acquisition and timed single-frame acquisition in synchronization with each one of the LEDs. The data were stored for reconstruction using separate software.

The geometry of the system included a pinhole, which acted as a size-limited effective light source placed at a distance of 6 cm above the image sensor ($z_{0}-z_{2}$). The distance from the sample to the sensor ($z_{1}-z_{2}$) was determined by the thickness of the petri dish bottom or glass carrier that contained the sample (normally between 0.5 to 1.5 mm). The system was placed inside a 3D-printed housing with a sufficiently large additional space to fit petri dishes of 9 cm in diameter (Figure 2b).

Microscope operation is possible both outside and inside a cell incubator environment. The setup has been tested long-term and has been proven to work for days both under air and room temperature and under standard cell incubator conditions (37 °C and 5% CO_2_).

### 2.3. Image Reconstruction and Examination

#### 2.3.1. Image Back Propagation

The data obtained by the DIHM described above contained a diffraction image of the illuminated sample on the image sensor. In order to reconstruct the sample, in-house written software utilizing the angular spectrum approach [17,18] was used.

The diffraction image ${\left|U_{z_{2}}\right|}^{2}$ is the intensity of the interference of the wave front O caused by diffraction at the sample and the illumination wave R itself (Equation (1)).

(1) $${\left|U_{z_{2}}\right|}^{2}={\left|R+O\right|}^{2}={\left|R\right|}^{2}+{\left|O\right|}^{2}+R\cdot O^{*}+R^{*}\cdot O$$

Here, ${\left|R\right|}^{2}$ is the intensity of the illumination wave (reference wave), ${\left|O\right|}^{2}$ is the zero-order diffraction of the sample, and $R^{*}\cdot O$ and $R\cdot O^{*}$ are the real image and the twin image, respectively. Only the real image and the twin image carry the desired sample information. The zero-order diffraction is typically small compared to the illumination intensity, and therefore normalizing ${\left|U_{z_{2}}\right|}^{2}$ to ${\left|R\right|}^{2}$ will reduce the dependency of the reconstruction on the illumination and the image sensor sensitivity [18] (Equation (2)).

(2) $${\left|{\tilde{U}}_{z_{2}}\right|}^{2}=\frac{{\left|U_{z_{2}}\right|}^{2}}{{\left|R\right|}^{2}}\approx 1+\frac{R\cdot O^{*}+R^{*}\cdot O}{{\left|R\right|}^{2}}$$

However, especially with moving samples and in the presence of a liquid medium, the reference wave intensity cannot be obtained easily by a measurement without a sample. Nevertheless, when the illumination wave can be approximated as a plane wave, this is not necessary. The error due to the divergent beam of a point light source results in a magnification factor between the reconstructed image and the real sample wave front [18]. Afterwards, the square root of the raw intensity, which gives the raw amplitude, is multiplied by the reference wave (a plane wave with normalized amplitude and constant phase), leading to $U_{0}$, and then Fourier transformed. The Fourier transformed wave front is then multiplied with the propagation function in Fourier space simulated for the optical distance (z/n) between the image sensor and the sample, and the result is back transformed into real space (Equation (3)).

(3) $$U_{z_{1}}\left(x_{1},y_{1}\right)=FT^{-1}\left\{FT\left\{U_{0}\left(x_{2},y_{2}\right)\right\}\exp\left(\frac{i2\pi zn}{\lambda }\cdot \sqrt{1-{\left(\frac{\lambda f_{x}}{n}\right)}^{2}-{\left(\frac{\lambda f_{y}}{n}\right)}^{2}}\right)\right\}$$

**Figure 3:**
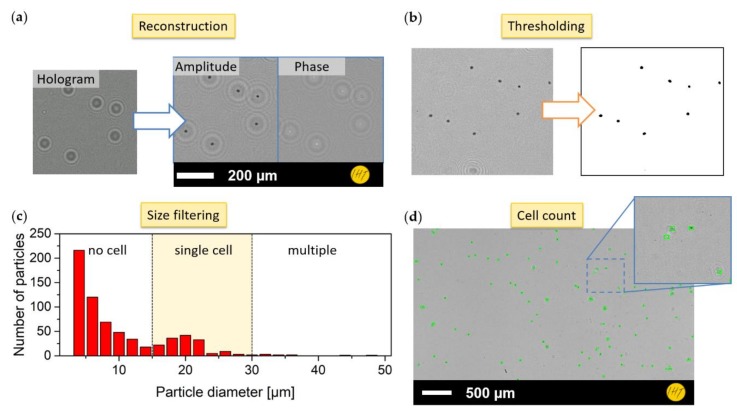
Sequential image processing steps for cell counting: (**a**) holographic reconstruction via angular spectrum approach; (**b**) image thresholding for background removal; (**c**) histogram of particles detected with blob detection and sorted by particle diameter; and (**d**) cell registration results with positive detected cells marked in green.

Here, λ is the wavelength of the light, n is the refractive index of the medium, z is the propagation distance, and $\left(x_{2},y_{2}\right)$, $\left(x_{1},y_{1}\right)$, and $\left(f_{x},f_{y}\right)$ are the coordinates in the $z_{2}$-plane, the $z_{1}$-plane, and Fourier space, respectively. The complex field $U_{z_{1}}$ gives both the amplitude and phase information of the wave front at the detector plane z_1_ (Figure 3a). For the reconstruction of video data, this approach is performed frame by frame.

Because of the differences in sample carriers, the distance from the image sensor to the sample plane might be unknown. To solve this, multiple small step reconstructions might be performed and the resulting image could be evaluated by an appropriate focus metric. In this work, the Gini of gradient (Gog) and the Tamura of gradient (Tog) were utilized [19,20], where the sample distance was given by a local maximum of the function.

#### 2.3.2. Still Frame Cell Counting

For cell counting, a blob detection approach based on connected pixels as part of the ImageJ suite [21,22,23] was utilized. In preparation for the blob detection, the intensity of the reconstructed image was filtered by a bandpass filter and then a threshold applied to generate a binary segmentation of image and background (Figure 3b). Then, the blob detection algorithm was applied, delivering a list of possible particle findings. This list, which gives particle size, area, and roundness, can subsequently be filtered according to a priori knowledge of the cells in culture (e.g., size range or area) (Figure 3c). After applying this filter, the resulting list of cells represents positively found objects. Figure 3d shows the found cells, which are marked in green on the original image.

#### 2.3.3. Video Particle Tracking

For video particle tracking, recordings with 7 fps were performed. In order to perform the particle tracking operation, ParticleTracker (MOSAIC suite) [24,25], which is an ImageJ plugin, was chosen because of its ease of use. The video sequence was reconstructed and extracted into single frames. To fit the requirements of ParticleTracker, the single frames were also inverted (dark to light) and the background level was eliminated via truncated threshold. The results of the tracker were exported and then further filtered by appropriate constraints.

## 3. Results

### 3.1. Microscopic Imaging Evaluation

**Figure 4:**
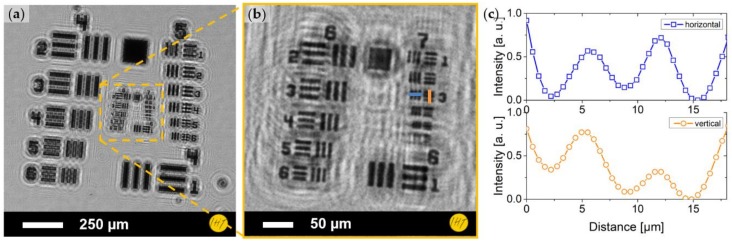
Holographic image of 1951 USAF resolution test chart: (**a**) view of Groups 4 and 5 of the chart; (**b**) enlarged view of Groups 6 and 7; and (**c**) normalized intensity profile through Element 3 of Group 7 (horizontally and vertically, see colored bars in (**b**)).

The imaging capability of the DIHM was evaluated by a standard resolution test target (1951 USAF resolution test chart, Figure 4a,b). The test structures were visible down to the third element of Group 7, which corresponds with a distance, as seen between the bars, of 3.1 µm. The normalized intensity plot is shown in Figure 4c.

### 3.2. Neuroblastoma Cells

**Figure 5:**
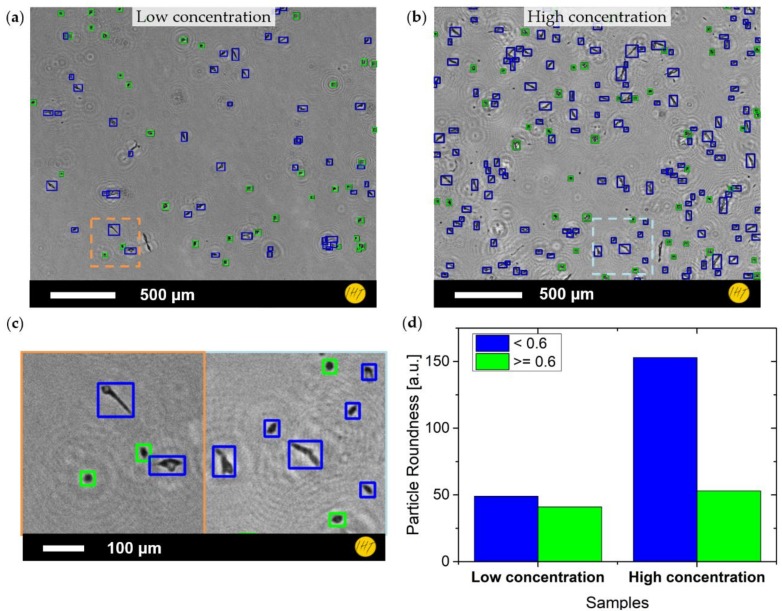
Cell registration of neuroblastoma cultures: (**a**) amplitude image of low concentration cell culture with cell findings overlay (blue for low roundness, green for high roundness); (**b**) amplitude image of high concentration cell culture with cell findings overlay (colors as in (**a**)); (**c**) zoomed-in images (orange and light blue square) showing the distinction between the two cell types; and (**d**) histogram of counts shown in (**a**,**b**) (matching colors).

Neuroblastoma cell cultures were investigated in two concentrations inside the wells of a well plate (i.e., sample low concentration and high concentration). The measurement was performed in air directly after taking the samples out of the incubator. Both single frame images and short video captures were made, with the video revealing both non-moving adherent cells and floating cells in the culture. Still images were taken with all three colors, though due to the absorption of the cultivation medium the illumination with the blue LED (466 nm peak) showed the best contrast. The measurements were reconstructed according to the angular spectrum approach described above. For statistical purposes, multiple positions on multiple recordings were investigated. Because the intensity images showed a clearer particle contrast then the phase images, they were used for the particle search via blob detection. The cells found have been visualized in Figure 5a,b, which correspond to low and high concentrations, respectively. Because of the irregular shape of the neuroblastoma cells, a simple cell filtering based on cell diameter could not be applied. Therefore, the filtering was carried out using cell area and ignoring smaller particles that might not be part of the cells. Furthermore, the cells showed different shapes: while some cells were round, others were elongated. This fact was used to classify the cell findings by a roundness factor [26] (Equation (4)).

(4) $$\mathrm{Roundness}=4\cdot \frac{\left[\mathrm{Area}\right]}{\pi \cdot {\left[\mathrm{Major}\;\mathrm{Axis}\right]}^{2}}$$

From a comparison of manual image observation with the automated cell counting results, roundness of ≈0.6 was considered a good dividing line between the two classes of cells. The cells found in the figures have been marked with blue boxes to indicate the less round category (<0.6) and green boxes to indicate the rounder one (≥0.6). Figure 5c shows the distinction between both types of cells, while Figure 5d depicts the quantitative count of the images shown in Figure 5a,b.

Multiple sample cutouts were used to determine the concentration of cells per area (Table 1). Furthermore, the segmented image was able to be used to calculate the surface coverage (confluence) of the culture.

The cell registration results were evaluated by randomly checking parts of the dataset through hand counting, suggesting a deviation of below 10%.

**Figure 6:**
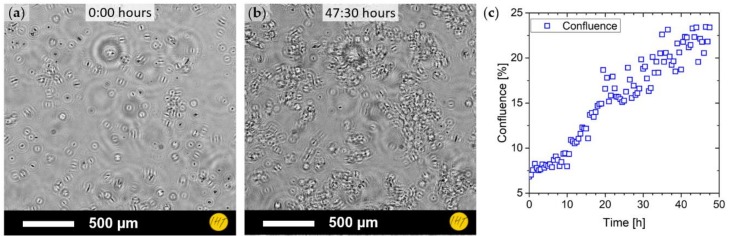
(**a**) Neuroblastoma cell culture measured inside a cell incubator shown at the start of the measurement period; (**b**) cell culture after two days; and (**c**) confluence estimation over the cultivation period.

In addition, the long-term growth of a neuroblastoma culture was monitored over two days inside a cell incubator. The cells were kept under 5% CO_2_ at a temperature of 37 °C. Images were taken every 30 min and the confluence of the culture was estimated for every measurement. DIHM images of the cell culture at the start of the experiment and after two days are shown in Figure 6a,b, respectively. The development of confluence is shown in Figure 6c, showing a significant growth in surface coverage by the cells. A time-lapse video of the measurement has been presented as Supplementary Materials (Video S1).

### 3.3. Prorocentrum Minimum

*P. minimum* cell cultures with three different cell densities, referred to as Samples 1 to 3, were been investigated. Samples 1, 2, and 3 had the lowest, medium, and highest density levels, respectively. The samples were measured in air directly after being taken out of the cell incubator. The images were recorded with the blue LED, which displayed the best contrast compared to the others.

**Figure 7:**
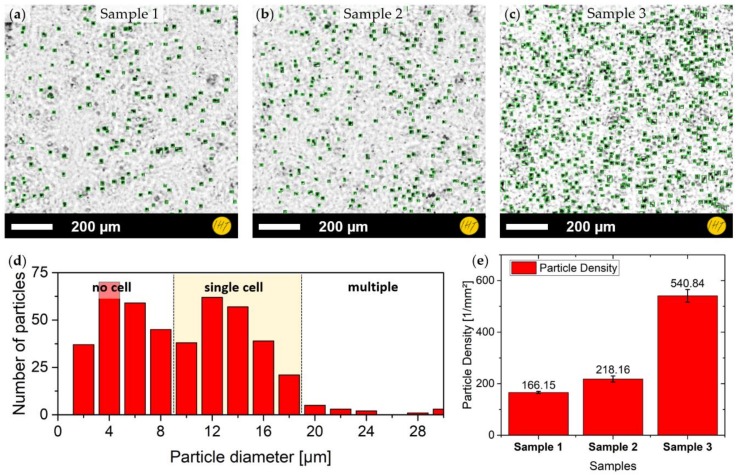
Cell counting for *Prorocentrum minimum*: (**a**–**c**) samples with three different cell concentrations, cell findings being marked with green boxes; (**d**) histogram of cell diameter for filtering the detected particles; and (**e**) particle densities for all three cell cultures.

Cell counting was performed for different positions in a larger image and at different time steps in the video. Example frames from each investigated cell density are shown in Figure 7a–c, with the cells having been found by blob detection and size filtering. A histogram of particle diameter is shown in Figure 7d. The distribution of cell sizes can be divided into three zones: (1) particles that are too small to be cells, (2) particles within the size distribution of single cells, and (3) particles that are too large to be a single cell and which might be multiple agglomerated cells. These cell classifications were defined by comparing the automated cell findings with hand-counted and classified data. The borders between the zones were adjusted to minimize the difference between both approaches.

The resulting cell counts were averaged and are shown in Figure 7e. When analyzing the video frames, cell movement can be seen. It appears that there are two kinds of cells: (1) those that are placed on the ground and are not moving and (2) cells that are floating through the medium. *P. minimum* cell registration was evaluated against hand counting, suggesting a mean deviation of about 12.5 ± 2.3%.

### 3.4. Cell Tracking

**Figure 8:**
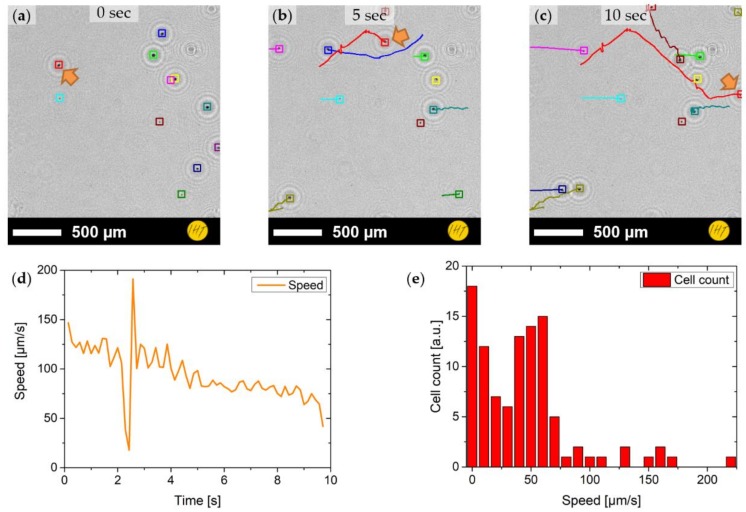
Cell tracking of *P. minimum* cells on a microscopy carrier: (**a**–**c**) tracking results from the start of the measurement (0 s) to 10 s with marked cell positions and trajectories; (**d**) extracted momentary speed of one cell trajectory (orange arrow in **a**–**c**); and (**e**) average speed distribution of all cells.

In order to confine the *P. minimum* cells to one layer, a drop of the cells in medium was dispensed onto a microscopy glass carrier and protected with a cover glass. This provided a flat layer of confined cells that could be easily tracked without the need for a three-dimensional reconstruction. Videos of up to 30 s were recorded and reconstructed as described above. The data were extracted as single frames, inverted, and binarized by means of thresholding. The identification and tracking of the cells are shown in Figure 8a–c at the start (0 s), at 5 s, and at 10 s, respectively. It can be demonstrated in the sample that two different types of cells are visible (i.e., cells that move quite actively and cells that mostly do not move and are in a more passive state). Furthermore, it can be seen that speed and direction of the cells can change over the course of the measurement. The momentary speed of one cell trajectory (orange arrow) is show in Figure 8d. A large variation in momentary speed can be observed. In order to obtain a general overview of the whole culture, the speed for every trajectory was averaged (Figure 8e). Here it can be seen that there is a large quantity of slow cells moving at speeds ranging 0–40 µm/s, followed by a similarly large quantity of cells moving at 40–80 µm/s. High velocities of above 80 µm/s only occur in small quantities.

## 4. Discussion

A DIHM, which is capable of continuously imaging both SH-SY5Y neuroblastoma and *P. minimum*, was achieved. Both cultures were measured inside standard cell containers like petri dishes and well plates. This suggests that further long-term investigation may not be reliant on special custom-made sample containers but could be carried out within standard culture equipment. A resolution down to 3.1 µm was able to be achieved and the quality of the imaging was sufficient for cell distinction.

When imaging the neuroblastoma cell culture, two distinct types of cells could be seen and classified: one type that is of oval or round shape and one type that is elongated and of irregular form. These types might correspond to viable and non-viable cells in the culture. For particle registration and counting, the irregularity of the shapes made simple filtering of the detected objects by size alone impractical. However, to sort cells from non-cell artifacts or impurities, an area constraint could be applied. In order to distinguish between two different cell types, an additional constraint, that evaluates the form of the found particles, was necessary. It has been shown that the particle roundness can serve as a constraint for the first approximation (see Figure 5c). However, pattern matching possibly in form of a neural network could give an easier and more reliable approach. Inside the wells of the well plate, both adherent and floating cells could be observed, while the holographic reconstruction, due to its limited depth resolution, could only show mostly a selective plane. Therefore, to evaluate the floating portion of the culture, further methods for a tomographic view of the sample need to be applied. Still, surface coverage of the bottom of the well could be easily obtained, especially with low-density samples. Nevertheless, long-term investigation of neuroblastoma cells was carried out over a measurement period of two days. This shows the capability of the developed setup to conduct long-term observations and indicates that it might provide continuous access to cell growth experiments.

*P. minimum* has been investigated regarding both cell density of adherent cells on the bottom of individual petri dishes and cell movement on microscopy slides. Because of the distinct size distribution of the *P. minimum* cells, particle filtering by size constraints was feasible and led to a good recognition of single algae while suppressing image artifacts and other residues. However, this method relies on both good quality images with a clear distinction between object and background and carefully adjusted filtering criteria. This makes full automation of this method challenging. Therefore, future work will evaluate the possibility of using a neural network approach that might be able to do self-adjustment.

## 5. Conclusions

In this study, a portable digital inline-holographic microscope (DIHM) has been proven to be a cost-effective and compact device for basic cell culture investigation. Due to its minimalistic setup, it is also robust in difficult environments. It is a promising option for long-term life-cell monitoring and kinetic tracking. Although in its basic setup, with resolution limited by the geometric magnification factor and the pixel pitch of the image sensor, for cell density investigation and particle tracking, low resolution might be sufficient. If higher resolution was required, pixel super-resolution techniques could be applied [27,28,29]. This, however, would limit the frame rate of the video imaging, making it undesirable for fast particles. The use of petri dishes and well plates makes the setup flexible, so that it can be applied to biological samples without the need for special preparation.

Future work will be focused on refining and calibrating the counting and tracking procedures. The incorporation of an artificial neural network for cell registration and distinction will be attempted. The limitation of reconstruction onto a single plane will be removed by full 3D reconstruction of the data.

## Figures and Tables

**Table 1:** Cell measurement results of neuroblastoma cell cultures.

	Low Concentration	High Concentration
Concentration [1/mm^2^]	15.2 ± 1.5	33.1 ± 2.1
Relation round/non-round [%]	52/48	26/74
Area coverage [%]	1.0	2.2

## Supplementary Materials

The following are available online at https://www.mdpi.com/1424-8220/19/5/1234/s1, Video S1: long_term_neuroblastoma.mp4.
